# High complexity of toxic reactions: parallels between products of oxidative stress and advanced glycation end products

**DOI:** 10.1007/s00204-020-02727-0

**Published:** 2020-04-01

**Authors:** Hermann M. Bolt

**Affiliations:** Department of Toxicology, Leibniz Research Centre for Working Environment and Human Factors at TU Dortmund (IfADo), Ardeystr. 67, 44139 Dortmund, Germany

In *Archives of Toxicology*, oxidative stress has been a matter of Editorials for years (Hengstler and Bolt 2007; Bolt and Hengstler 2010a; Stewart et al. 2011; Bolt and Stewart 2012; Marchan 2015). High citation numbers underline its scientific impact (Bolt and Hengstler 2010b). In our recent March issue, Gulcin (2020) presents a new comprehensive update on antioxidants and associated methodologies. Among others, Gulcin points to an analogy that is mostly overlooked. He outlines that oxidative stress produces reactive carbonyls and associated reaction products which may be referred to as *advanced lipid oxidation products*, by analogy to *advanced glycation*^1^*end products* (AGEs).

In food toxicology, AGEs are long known to the result from the *Maillard* reaction (Maillard 1912) during heat processing of foods. With recent progress in biochemistry and analytical chemistry (Distler et al. 2014), this scientific field is currently receiving pronounced interest (Delgado-Andrade and Fogliano 2018). AGEs are non-enzymatic protein and amino acid adducts which are formed from carbohydrate-derived dicarbonyls. There is also endogenous AGE formation, which is enhanced in diabetes mellitus. This is considered to be associated with the development of diabetic complications (Brings et al. 2017) and age-related diseases in general (Rowan et al. 2018). In the eye lens, both protein glycation (Bejarano and Taylor 2019) and oxidative stress (Ma et al. 2019; Wojnar et al. 2020) are thought to play significant roles in cataract development. The chemical complexity of heat-induced food contaminants is also a matter of in silico toxicological studies (Frenzel et al. 2017).

Furthermore, the formation of AGEs resulting from carbohydrate degradation has also been addressed as a problem in drug safety, e.g. in heat sterilization of glucose-containing infusion fluids (Schalwijk et al. 1999; Pischetsrieder et al. 2016).

The parallelism between products of oxidative stress and advanced glycation end products, together with associated methodologies, points to new ways for research into the biological complexity of endogenous and exogenous toxicants. Contributions to this emerging field are therefore highly welcome to *Archives of Toxicology*.

## Footnotes

1 Some authors speak of “advanced glycosylation end products”, others of “advanced glycation end products”.
